# Spread of Terbinafine-Resistant *Trichophyton mentagrophytes* Type VIII (India) in Germany–“The Tip of the Iceberg?”

**DOI:** 10.3390/jof6040207

**Published:** 2020-10-05

**Authors:** Pietro Nenoff, Shyam B. Verma, Andreas Ebert, Anke Süß, Eleni Fischer, Elke Auerswald, Stephanie Dessoi, Wencke Hofmann, Simone Schmidt, Kathrin Neubert, Regina Renner, Sirius Sohl, Uta Hradetzky, Ursula Krusche, Hans-Christian Wenzel, Annegret Staginnus, Jörg Schaller, Valentina Müller, Christiane Tauer, Matthias Gebhardt, Katja Schubert, Zaid Almustafa, Rudolf Stadler, Andrea Fuchs, Cassian Sitaru, Carsten Retzlaff, Cora Overbeck, Thomas Neumann, Anette Kerschnitzki, Stephan Krause, Martin Schaller, Birgit Walker, Thomas Walther, Lars Köhler, Manuela Albrecht, Ursula Willing, Michel Monod, Karine Salamin, Anke Burmester, Daniela Koch, Constanze Krüger, Silke Uhrlaß

**Affiliations:** 1 Labor für Medizinische Mikrobiologie, 04571 Rötha OT Mölbis, Germany; andebe47@gmail.com (A.E.); koch@mykologie-experten.de (D.K.); krueger@mykologie-experten.de (C.K.); S.Uhrlass@mykologie-experten.de (S.U.); 2 “Nirvan” and “In Skin” Clinics, Vadodara, 390020 Gujarat, India; skindiaverma@gmail.com; 3 Medizinische Fakultät, Universität Leipzig, 04103 Leipzig, Germany; 4 Hautärztin, Gemeinschaftspraxis Allgemeinmedizin und Dermatologie, Kurfürstenstraße 23 a, 54616 Wittlich, Germany; anke.suess@gmx.net; 5 Hautarztpraxis Gunhild Kratzsch, Kochstraße 66, 04275 Leipzig, Germany; evalerkou@hotmail.com; 6 Hautarztpraxis, Lungwitzer Straße 30, 09337 Hohenstein-Ernstthal, Germany; e.auerswald@online.de; 7 Hautzentrum Nordwest, Tituscorso 2-6, 60439 Frankfurt am Main, Germany; praxis@hautzentrum-nordwest.de (S.D.); info@hautzentrum-nordwest.de (W.H.); 8 Hautarztpraxis im MVZ Friedrichstadt, Friedrichstraße 41, Haus A-Haupteingang-1. OG, 01067 Dresden, Germany; s.schmidt.derma@gmail.com; 9 Hautarztpraxis Dipl. med. Kathrin Neubert, Bertolt- Brecht-Straße 2, 09217 Burgstädt, Germany; PraxisKNeubert@t-online.de; 10 Gemeinschaftspraxis Dres. med. S. Sohl & R. Renner, Katharinenstr. 33, 73728 Esslingen, Germany; info@hautarztpraxis-esslingen.de (R.R.); sohl@hautarztpraxis-esslingen.de (S.S.); 11 Hautarztpraxis, Landsberger Straße 4, 04157 Leipzig, Germany; uta.hradetzky@yahoo.de (U.H.); nenoff@mykologie-experten.de (U.K.); 12 Hautarztpraxis/Phlebologie, Waldkerbelstraße 12, 04329 Leipzig, Germany; hc.wenzel@vz-bruehl.de; 13 Hautarztpraxis, 04668 Grimma, Germany; staginnus@email.de; 14 Dermatopathologie Duisburg, J. Schaller und C. Hendricks, 47166 Duisburg, Germany; dermpathdu@aol.com; 15 Helios Klinikum Duisburg, Klinik für Dermatologie, Allergologie und Phlebologie, 47166 Duisburg, Germany; va.mueller@gmx.de; 16 Hautarztpraxis Christiane Tauer, Marcel- Callo- Platz 4, 98544 Zella-Mehlis, Germany; christianetauer@gmx.de; 17 Hautarztpraxis, Matthias Gebhardt, Leipziger Str. 90, 08058 Zwickau, Germany; gebderm@t-online.de; 18 Hautarztpraxis Dresden, Loschwitzer Straße 40, D-01309 Dresden, Germany; kontakt@meine-hautaerztin.de; 19 Klinik für Dermatologie, Charite, 10117 Berlin, Germany; zalmustafa@gmail.com; 20 Department of Dermatology, Qatif Central Hospital, Al Qatif 32654, Saudi Arabia; 21 Universitätsklinik für Dermatologie, Venerologie, Allergologie und Phlebologie, Johannes Wesling Klinikum Minden, Hans-Nolte-Straße 1, D-32429 Minden, Germany; rudolf.stadler@t-online.de; 22 Hautarztpraxis, Darwinstr. 1, 04600 Altenburg, Germany; andrea.-fuchs@web.de; 23 Universitätsklinikum Freiburg, Klinik für Dermatologie und Venereologie, Mykologisches Labor, Hauptstraße 7, 79104 Freiburg, Germany; cassian.sitaru@mail.medizin.uni-freiburg.de; 24 Labor Dres. Löbel und Retzlaff, Felsbachstr. 5, 07745 Jena, Germany; Carsten.Retzlaff@amedes-group.com; 25 Gemeinschaftspraxis Dermatologie Cora Overbeck und Thomas Neumann, Lange Str. 53, 34346 Hann. Münden, Germany; cora.overbeck@t-online.de (C.O.); thneu@icloud.com (T.N.); 26 Klinik für Dermatologie, Klinikum Uni München, Mykologisches Labor, Frauenlobstr. 9-11, 80337 München, Germany; Anette.Kerschnitzki@med.uni-muenchen.de; 27 Stephan Krause, Bundeswehrkrankenhaus Berlin, Klinik III Dermatologie, Scharnhorststraße 13, 10115 Berlin, Germany; stephan2krause@bundeswehr.org; 28 Department of Dermatology, University Clinic Tübingen, Liebermeisterstraße 25, 72076 Tübingen, Germany; martin.schaller@med.uni-tuebingen.de (M.S.); Birgit.Walker@med.uni-tuebingen.de (B.W.); 29 Thomas Walther, Hautarztpraxis, Bästleinstr. 6, 04347 Leipzig, Germany; familie.dr.walther@gmail.com; 30 Hautarztpraxis, Boppstr. 20-24, 55118 Mainz, Germany; hautarzt-koehler@web.de; 31 Hautarztpraxis, Mockauer Str. 123-125, 04357 Leipzig, Germany; albm@gmx.de (M.A.); info@hautaerztin-wurzen.de (U.W.); 32 Dermatology Service, Centre Hospitalier Universitaire Vaudois, 1011 Lausanne, Switzerland; Michel.Monod@chuv.ch (M.M.); Karine.Salamin@chuv.ch (K.S.); 33 Department of Dermatology, University Hospital Jena, Erfurter Str. 35, D-07743 Jena, Germany; Anke.Burmester@med.uni-jena.de

**Keywords:** dermatophytoses, terbinafine-resistant, squalene epoxidase, point mutation, transmission, Itraconazole, Ciclopirox, Miconazole

## Abstract

Chronic recalcitrant dermatophytoses, due to *Trichophyton* (*T*.) *mentagrophytes* Type VIII are on the rise in India and are noteworthy for their predominance. It would not be wrong to assume that travel and migration would be responsible for the spread of *T*. *mentagrophytes* Type VIII from India, with many strains resistant to terbinafine, to other parts of the world. From September 2016 until March 2020, a total of 29 strains of *T*. *mentagrophytes* Type VIII (India) were isolated. All patients were residents of Germany: 12 females, 15 males and the gender of the remaining two was not assignable. Patients originated from India (11), Pakistan (two), Bangladesh (one), Iraq (two), Bahrain (one), Libya (one) and other unspecified countries (10). At least two patients were German-born residents. Most samples (21) were collected in 2019 and 2020. All 29 *T*. *mentagrophytes* isolates were sequenced (internal transcribed spacer (ITS) and translation elongation factor 1-α gene (TEF1-α)). All were identified as genotype VIII (India) of *T*. *mentagrophytes*. In vitro resistance testing revealed 13/29 strains (45%) to be terbinafine-resistant with minimum inhibitory concentration (MIC) breakpoints ≥0.2 µg/mL. The remaining 16 strains (55%) were terbinafine-sensitive. Point mutation analysis revealed that 10/13 resistant strains exhibited Phe^397^Leu amino acid substitution of squalene epoxidase (SQLE), indicative for in vitro resistance to terbinafine. Two resistant strains showed combined Phe^397^Leu and Ala^448^Thr amino acid substitutions, and one strain a single Leu^393^Phe amino acid substitution. Out of 16 terbinafine-sensitive strains, in eight Ala^448^Thr, and in one Ala^448^Thr +, new Val^444^ Ile amino acid substitutions were detected. Resistance to both itraconazole and voriconazole was observed in three out of 13 analyzed strains. Treatment included topical ciclopirox olamine plus topical miconazole or sertaconazole. Oral itraconazole 200 mg twice daily for four to eight weeks was found to be adequate. Terbinafine-resistant *T*. *mentagrophytes* Type VIII are being increasingly isolated. In Germany, transmission of *T*. *mentagrophytes* Type VIII from the Indian subcontinent to Europe should be viewed as a significant public health issue.

## 1. Introduction

There is a veritable epidemic of varieties of chronic recalcitrant dermatophytoses due to *Trichophyton* (*T*.) *mentagrophytes* Type VIII in India [1]. A wide variation in clinical features is seen. Tinea corporis, tinea cruris, tinea faciei and their combinations are the most common presentations. Lesions often show a minimal to a high degree of inflammation, and large lesions with a tendency to coalesce and spread are common. Severe itching is common [2]. There has been an undeniable association between the occurrence of extensive and hard to treat tinea and long-term abuse of potent and super-potent topical corticosteroids, predominantly clobetasol propionate [1,3].

Extensive travel and migration are considered vital in the spread of dermatophytoses. Especially the terbinafine-resistant strains of *T*. *mentagrophytes* Type VIII, detected as the causative genotype, are now increasingly isolated in Germany and other European countries. The main criterion to identify this particular infection is the very noticeable treatment failure with topical and oral terbinafine.

## 2. Patients and Methods

### 2.1. Patients

Patients discussed herein were predominantly German residents with chronic dermatophytoses who had been failing treatment with terbinafine and, therefore, were suspected to harbour *T*. *mentagrophytes* Type VIII. They were subjected to mycological diagnostics including Blancophor^®^ preparation, fungal culture and molecular biological fungal DNA detection. Skin scrapings taken from suspicious skin sites were investigated. Additionally, a few fungal cultures isolated in other laboratories were sent to our center for precise identification of the fungal species and internal transcribed spacer (ITS) genotype has been included in the epidemiological investigation (Table 1).

### 2.2. Conventional Cultural Diagnostics

In mycological routine diagnostics, scrapings from the active edges of centrifugally spreading lesions of the free skin, as well as hair roots from lesions of the capillitium in some patients, were cultured on Sabouraud´s 4% dextrose agar (Sifin, Berlin, Germany) and, additionally, on cycloheximide (Actidione^®^-containing Sabouraud´s dextrose agar, Becton Dickinson, Heidelberg, Germany). Fungal isolates showing fast-growing, flat radiating fungal colonies with a white periphery, and sometimes bright yellowish centre typical for *T*. *mentagrophytes*, were further analyzed. Microscopic lactophenol cotton blue preparations were performed from such colonies.

### 2.3. Molecular Biological Characteristics of Trichophyton mentagrophytes ITS Type VIII

#### 2.3.1. PCR-ELISA for Molecular Identification of Dermatophytes

DNA from either skin scrapings or fungal isolates (for identification of submitted fungal cultures) was extracted according to the manufacturer´s protocol using the QIAamp^®^ DNA Mini Kit (Qiagen, Hilden, Germany). Samples were analyzed using a validated and standardized in-house developed enzyme linked immunoassay (PCR-ELISA) to detect dermatophyte DNA [4,5]. Specific probes detecting the following relevant dermatophytes were used: *T. rubrum*, *T*. *interdigitale*/*T*. *mentagrophytes*, *Microsporum canis*, and *T. benhamiae* (formerly referred to as *T. anamorph* or *Arthroderma benhamiae*).

All DNA samples extracted either from skin scrapings or from fungal cultures were positive in the PCR-ELISA for *T*. *interdigitale*/*T*. *mentagrophytes*. As the differentiation between *T*. *interdigitale* and the *T*. *mentagrophytes* complex was not possible by PCR-ELISA, the ITS regions of rDNA genes and the translation elongation factor (TEF)1-α gene were sequenced.

#### 2.3.2. Sequencing of the ITS Regions of rDNA Genes for Species Identification of *Trichophyton mentagrophytes* Type VIII

For confirmation of the suspected dermatophyte species, Sanger sequencing of the ITS regions of rDNA genes (mainly the regions ITS 1, 5.8 S rRNA, ITS 2) and *TEF1-α* gene was performed for all isolates [6,7,8,9]. This required PCR amplification of a ∼900 bp DNA fragment using universal primers that bind to flanking pan-fungal sequence regions: V9G (5’-TTACGTCCCTGCCCTTTGTA-3’) and LS266 (5’-GCATTCCCAAACAACTCGACTC-3’).

The length of the analyezd region in the *TEF1-α* gene varied from 709 to 769 nucleotides among the various dermatophyte species. Primers EF1a-F (5’-CACATTAACTTGGTCGTTATCG-3’) and EF1a-R (5’-CATCCTTGGAGATACCAGC-3’) were used for sequencing [6].

#### 2.3.3. Phylogenetic Analysis of *Trichophyton mentagrophytes* Type VIII

Both reference strains and clinically isolated wild type strains were used for comparative molecular analysis, and the generation of the phylogenetic tree based on the ITS region and the TEF 1α gene is listed in Table 2. In addition, GenBank numbers of all sequences used for generating phylogenetic trees are provided in Table 2.

#### 2.3.4. Statistical Method for Generating Phylogenetic Trees

Phylogenetic relationships between dermatophyte species were generated using the software Mega X: Analysis Statistical Method Maximum Likelihood, Phylogeny Test Bootstrap Method Replications–1000, Substitution Model Maximum Composite Likelihood [10,11].

#### 2.3.5. Deposition of the Isolates in Strain Collections and Gene Databases

Both ITS and TEF1 α gene sequences of all 29 strains/isolates are deposited at the database of the National Centre for Biotechnology Information (NCBI) in Bethesda, MD, USA (Table 1). The strains themselves were deposited at the German Collection of Microorganisms and Cell Cultures (DSMZ, Braunschweig, Germany).

### 2.4. Antifungal Resistance Testing

#### 2.4.1. In Vitro Antifungal Susceptibility Testing

Isolated dermatophytes growing on culture media were tested for growth on Sabouraud’s dextrose agar containing 0.2 µg/mL terbinafine, as described by previous research [12]. The concentration of terbinafine was equivalent to twice that of the minimal inhibitory concentration (MIC) for *T*. *mentagrophytes* and *T*. *rubrum* under these conditions [13]. Fungal growth was examined after seven and 14 days. Growing strains were recorded as resistant. MICs of itraconazole and voriconazole were determined according to the broth microdilution method of the Clinical and Laboratory Standards Institute as previously described [14]. Based on epidemiological cut-off values (ECOFFs) from previous research, strains were classified as resistant or sensitive to itraconazole and voriconazole (ECOFF ≥ 0.5 µg/mL for itraconazole; ECOFF ≥ 0.25 µg/mL for voriconazole) [15].

#### 2.4.2. Squalene Epoxidase Gene Analysis

Trichophyton total DNA was extracted from fresh fungal cultures on Sabouraud´s dextrose agar using a DNeasy Plant Minikit (Qiagen, Hilden, Germany). A square shaped area of approximately 1.0 mm^2^ of growing culture was used. The squalene epoxidase (*SQLE*) gene of the terbinafine-resistant clinical isolates was amplified by PCR with ReadyMix Taq PCR Reaction Mix (Sigma Aldrich, Merck). The primer pair TrSQLE-F2 (5’ ATGGTTGTAGAGGCTCCTCCC 3’) and TrSQLE-R1 (5’ CTAGCTTTGAAGTTCGGCAAA 3’) was used and chromosomal DNA served as the template. In some cases where fungal cultures were not obtained, *SQLE* gene fragments were analyzed from scale DNA as described [16] using primer pairs TmSQLEF4 (5’ AACGGCTTTGCGAATGGCTCC 3’) and TmSQLER4 (5’ GATGACCCTGCAGGCAGTAAG 3’). Sequences were aligned and screened for missense mutations using MEGA version 10.0.5 [10,11].

## 3. Results

### 3.1. Patients

Twenty-nine patients (all out-patients) with different clinical variants of dermatophytoses caused by *T*. *mentagrophytes* Type VIII (India) were diagnosed all over Germany (Table 1) between September 2016 and March 2020. The detection was based on both routine diagnostics performed in the laboratory Mölbis, Germany, and from cultures sent for fungal species identification of *T*. *mentagrophytes* Type VIII (India). The microbiological as well as molecular diagnosis of *T*. *mentagrophytes* Type VIII was possible in all 29 patients.

**Figure 1:**
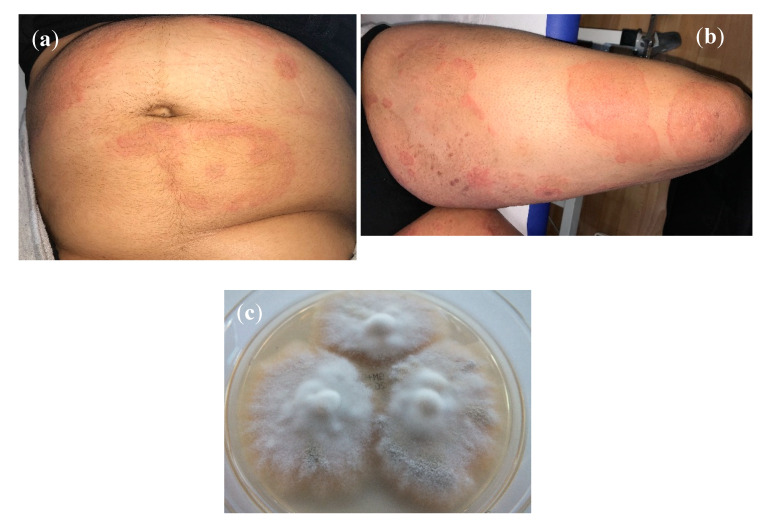
(**a**) Tinea corporis due to *T*. *mentagrophytes* VIII in a 34-year old female from India residing in Germany. Patient No. 12. Laboratory number of the fungal isolate: 600174/2019. (**b**) Accompanying large area tinea of the thighs of the same patient. (**c**) Subculture of *T. mentagrophytes* VIII isolated from the patient´s skin scrapings on Sabouraud´s dextrose agar; developed fast growing white, flat, radiating and granular colonies.

Males (*n* = 15) outnumbered females (*n* = 12). Gender was not specified for two patients. Patients’ age ranged from six months to 58 years. The mean age was 26 years (*n*= 26, for three patients the age was not known). Patients included two children aged six months and four years. Tinea corporis was the predominant variant of dermatophytosis (*n* = 14). Other variants of dermatophytoses were unspecified tinea (*n* = 3), tinea corporis and tinea cruris (*n* = 3), tinea cruris (*n* = 2), tinea corporis (including tinea glutealis) (*n* = 2), tinea faciei (*n* = 2), tinea corporis and tinea unguium (*n* = 1), tinea pedis (*n* = 1), and tinea corporis and tinea manuum (*n* = 1) (Table 1, Figure 1a,b). Both toddlers presented with tinea corporis (including tinea glutealis).

### 3.2. Some Striking Clinical Presentations

**Figure 2:**
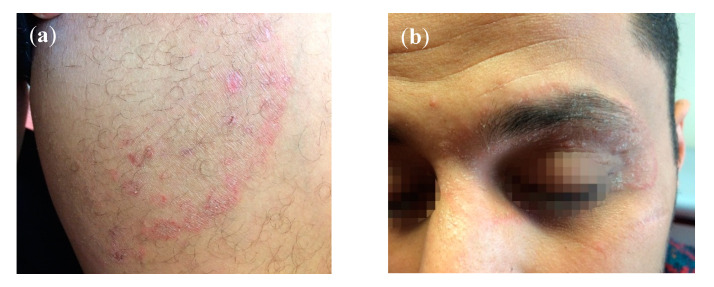
(**a**) Tinea cruris due to *T*. *mentagrophytes* VIII in a 28-year old male from Libya residing in Germany for about three years. Patient No. 10. Laboratory number of the fungal isolate: 202953/2019. (**b**) Accompanying tinea faciei of the same patient involving the left upper and lower eyelids.

**Figure 3:**
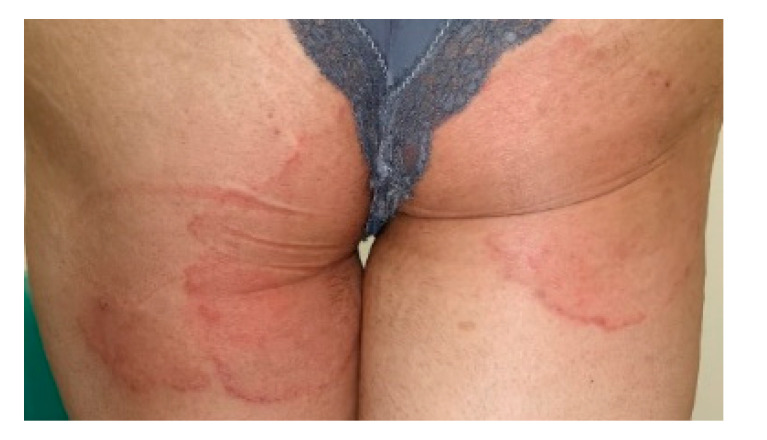
Tinea corporis und tinea glutealis due to *T*. *mentagrophytes* VIII in a woman originating from Iraq and living for a long time in Germany. Typical inflammatory and itching erythematosquamous plaques were observed involving the buttocks, the groin and the thighs. Patient No. 14. Laboratory number of the fungal isolate: 205667/2019.

(1) A six months-old-female infant from Bahrain visiting Germany with her family for a holiday was seen by us for extensive dermatophytosis of the back, buttocks, chest and groin [17]. Topical treatment by terbinafine for over two months was unsuccessful. Other family members, including adults and children, were treated in Bahrain with topical antifungals and oral voriconazole which was not helpful. The girl was successfully treated by topical miconazole and later by ciclopirox olamine.

(2) A 28-year old male from Libya, living for three years in Germany, suffered from tinea cruris and tinea faciei involving the left upper and lower eyelids (Figure 2a,b). Treatment by oral fluconazole and terbinafine had failed. His German girlfriend was also affected by the dermatophytosis, though her child was spared. The patient had no contact with India, Indians or Arabs and had not visited Libya in the past few years. The man, however, regularly went to the gym. Treatment with itraconazole orally 400 mg daily for eight weeks cured him.

(3) A pregnant German woman presented with tinea cruris et corporis after a trip to Saudi Arabia. Her husband was also affected. Topical treatment was started by clotrimazole alone.

(4) An Iraqi couple living in Germany for a long time suffered from chronic recalcitrant dermatophytosis of the groin, thighs and buttocks for at least two years (Figure 3). Repeated topical treatments by fixed-dose combination creams (FDCs), also known as combination creams, (fluprednidene 21-acetate + miconazole nitrate, betamethasone dipropionate + gentamicin sulphate, and betamethasone dipropionate + clotrimazole) had failed. Topical antifungal therapy (ciclopirox olamine, sertaconazole) given for five to six weeks acted very slowly and they stopped treatment due to progress of the disease. Oral itraconazole 200 mg daily was started for four weeks leading to resolution.

Phylogenetic analysis of Trichophyton mentagrophytes Type VIII in comparison to other genotypes. 

**Figure 4:**
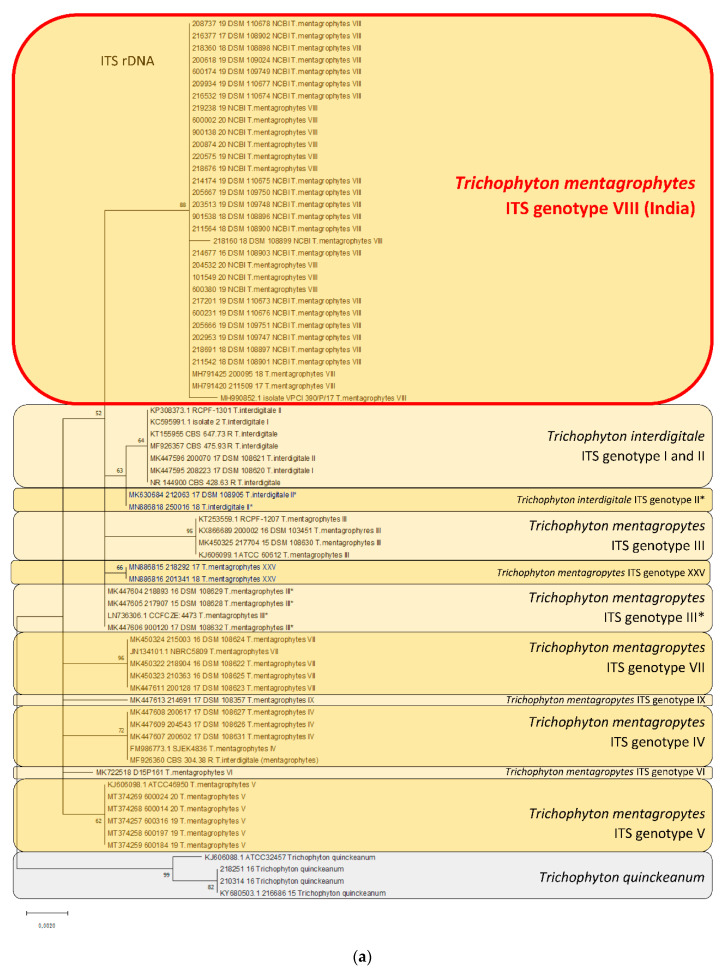

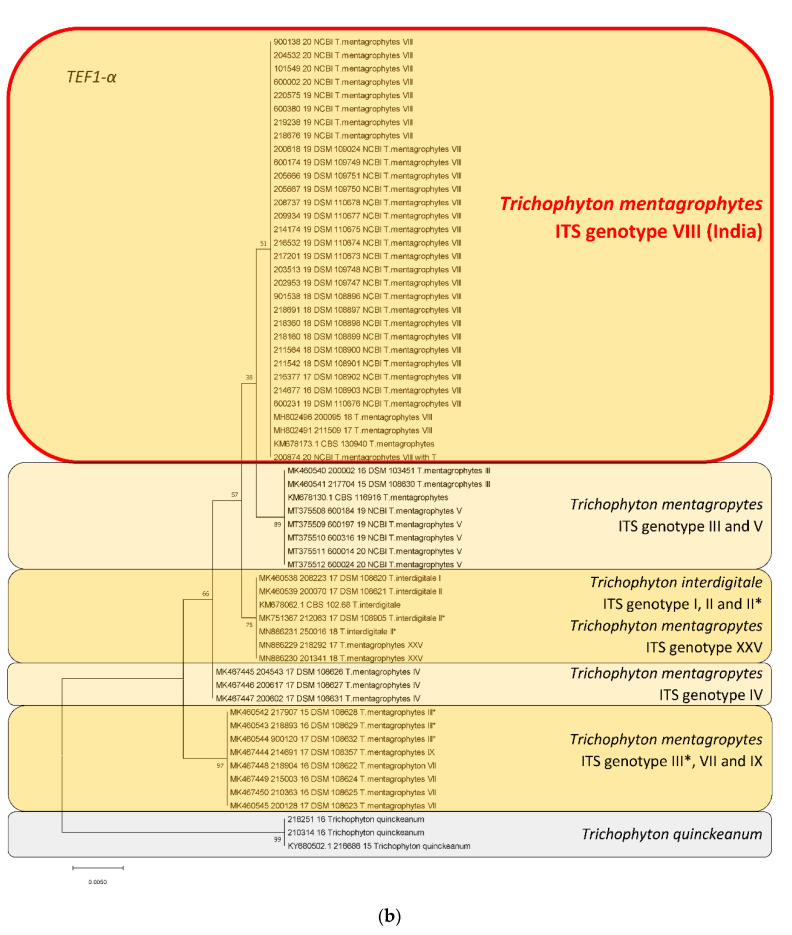
Phylogenetic analysis of the dermatophyte isolates based on the ITS regions of rDNA and the *TEF1-α* gene (Table 2).The evolutionary history was inferred by using the maximum likelihood method and Tamura-Nei model [10]. The tree with the highest log likelihood (−907.70) is shown. The percentage of trees in which the associated taxa clustered together is shown next to the branches. Initial tree(s) for the heuristic search were obtained automatically by applying Neighbor-Join and BioNJ algorithms to a matrix of pairwise distances estimated using the maximum composite likelihood (MCL) approach, and then selecting the topology with superior log likelihood value. The tree is drawn to scale, with branch lengths measured in the number of substitutions per site. This analysis involved 28 nucleotide sequences. Codon positions included were 1st + 2nd + 3rd + noncoding. There were a total of 1086 positions in the final dataset. Evolutionary analyses were conducted in MEGA X [11]. (**a**) Phylogenetic tree of *T*. *mentagrophytes* based on sequencing of the ITS regions of rDNA genes. By sequencing, a 100% concordance with NCBI reference strains (accession numbers MH791420, MH791425, MH990852) was found for all 29 isolates. All these isolates formed their own cluster, which is now called the ITS genotype VIII (India) of *T*. *mentagrophytes*. These isolates (*T*. *mentagrophytes* Type VIII or clade) were clearly discriminated from already known *T*. *mentagrophytes* genotypes, e.g., II, V, VII. Rooted with *Trichophyton quinckeanum*. (**b**) Phylogenetic tree of *T*. *mentagrophytes* based on sequencing of the *TEF1-α* gene. Used NCBI reference sequences (*TEF1-α* gene) were MH802491 and MH802496 (accession numbers). Within the phylogenetic tree, all *T*. *mentagrophytes* ITS Type VIII strains from Germany formed their own clade, which is clearly discriminated from the other, above mentioned, *T*. *mentagrophytes* genotypes. Rooted with *T. quinckeanum*.

Species identification was confirmed for all 29 isolates by sequencing of the ITS regions of rDNA genes. Molecular relationships of these 29 isolates with other genotypes within the species *T*. *mentagrophytes*, and with closely related dermatophytes, were depicted in a phylogenetic tree/dendrogram of the sequences (Figure 4a). All 29 isolates belonged to the same cluster, called ITS Type VIII, referred to as Indian variant. The isolates of *T*. *mentagrophytes* ITS Type VIII formed their own phylogenetic cluster. This genotype was clearly different from other already known genotypes of *T*. *mentagrophytes*, e.g., zoophilic strains isolated from human dermatophytoses and from animals, including a snow leopard at a zoo garden, and from *T*. *mentagrophytes* ITS genotype VII (Thai variant). The anthropophilic *T*. *interdigitale* could be distinguished clearly from zoophilic *T*. *mentagrophytes* clusters.

The phylogenetic tree based on sequencing of the *TEF1-α* gene revealed a 100% concordance of all 29 isolates belonging to genotype VIII of *T*. *mentagrophytes* (Figure 4b). Within the phylogenetic tree, all *T*. *mentagrophytes* isolates from this study formed their own clade, which was clearly differentiated from other *T*. *mentagrophytes* genotypes, and from *T*. *interdigitale*.

The origin of all reference strains, and clinical or animal isolates and their sequences used here for comparison, is presented in Table 2.

### 3.3. Antifungal Resistance Testing and Point Mutation Analysis

In vitro resistance testing revealed that 13 (45%) out of 29 strains were terbinafine-resistant with breakpoints ≥ 0.2 µg/mL. The remaining 16 strains (55%) were terbinafine-sensitive (Table 1). Point mutation analysis revealed that among 13 resistant strains, 10 exhibited Phe^397^Leu amino acid substitution of the SQLE, indicative of in vitro resistance to terbinafine. Two resistant strains showed combined Phe^397^Leu and Ala^448^Thr amino acid substitutions, and one strain a single Leu^393^Phe amino acid substitution. Out of 16 terbinafine-sensitive strains, Ala^448^Thr was detected in nine strains, one of which also showed a new Val^444^Ile substitution. The remainder of the sensitive strains exhibited no substitution. Out of 13 strains tested for triazole sensitivity, nine proved to be sensitive to both itraconazole and voriconazole. Three strains revealed resistance to both triazoles, with one strain also showing resistance to terbinafine, while the remaining strain exhibited resistance to voriconazole but not itraconazole. The accession numbers of sequences of the SQLE gene of all 29 investigated *T*. *mentagrophytes* strains are deposited at the NCBI database (Table 1).

## 4. Discussion

### 4.1. Trichophyton mentagrophytes Type VIII (India)

ITS genotype VIII of *T*. *mentagrophytes* seems to have grown rapidly in recent years to become the predominant dermatophyte in India [18]. In contrast, the previously predominant *T*. *rubrum* for decades was isolated with much less frequency [19]. A currently published epidemiological study of a total of 402 Indian patients with extensive dermatophytoses revealed culture growth of *T*. *mentagrophytes* in 289 (71.9%) of samples [15]. *T. rubrum* was cultivated from only 19 (4.7%) samples. It was possible to identify *T*. *interdigitale*/*T*. *mentagrophytes* complex in 235/265 (88.7%) of samples by PCR-ELISA. DNA sequencing enabled identification of *T. mentagrophytes* ITS Type VIII in 311 (77%) samples, unspecified species of *T*. *interdigitale*/*T*. *mentagrophytes* complex in 21 (5%), and *T. rubrum* in 19 (5%) samples.

**Figure 5:**
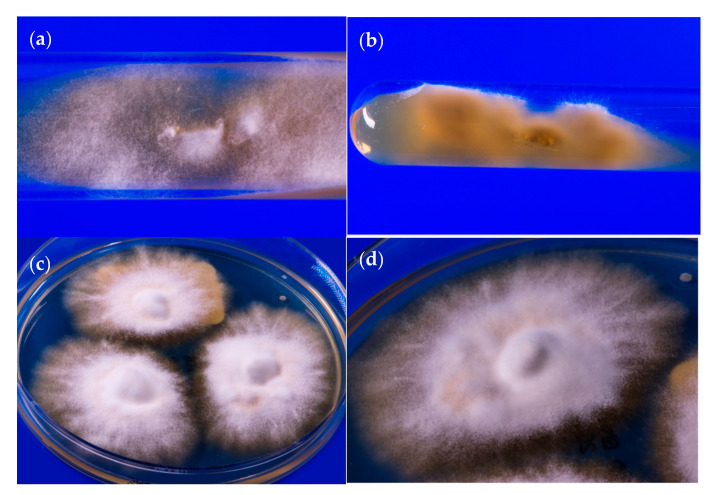
58-year old male originating from India suffering from tinea faciei due to *T*. *mentagrophytes* Type VIII. (**a**) Primary culture on slant agar tubes containing Sabouraud´s dextrose agar. Granular white, flat, fast-growing fungal colonies. (**b**) Yellow to brown stained reverse side of colonies on slant agar tubes. (**c**) Subculture of *T*. *mentagrophytes* Type VIII on Sabouraud´s dextrose agar petri dish agar. White, radiating and granular colonies with slightly yellowish stained centre of the colonies. (**d**) Single colony of *T*. *mentagrophytes* Type VIII on Sabouraud´s dextrose agar petri dish.

It is interesting to note that *T. mentagrophytes* Type VIII was not initially found in India, but was previously isolated in Oman, Iran and also in Australia under a different species name, *T*. *interdigitale*, in accordance with the old taxonomy of dermatophytes that was valid only until 2016 [20,21,22,23]. Currently, the species found in India and several other countries globally is referred to as *T*. *mentagrophytes* Type VIII in the dermatomycological community [24]. *T. mentagrophytes* Type VIII is only one variety within the cluster of a large number of genotypes of the *T*. *mentagrophytes*/*T*. *interdigitale* complex [16,21,25,26,27,28,29,30] (Figure 5a–d). Therefore, it does not appear justifiable to attempt assigning this single genotype VIII of *T*. *mentagrophytes* to a brand new species, so hurriedly, on the basis of just two isolated terbinafine-resistance isolates of *T*. *mentagrophytes*, disregarding the plethora of literature on a well-accepted taxonomy and nuances of this genotype, as has just happened in an isolated publication from Japan [31].

### 4.2. Spread of T. mentagrophytes Type VIII (India) to Other Parts of the World

**Figure 6:**
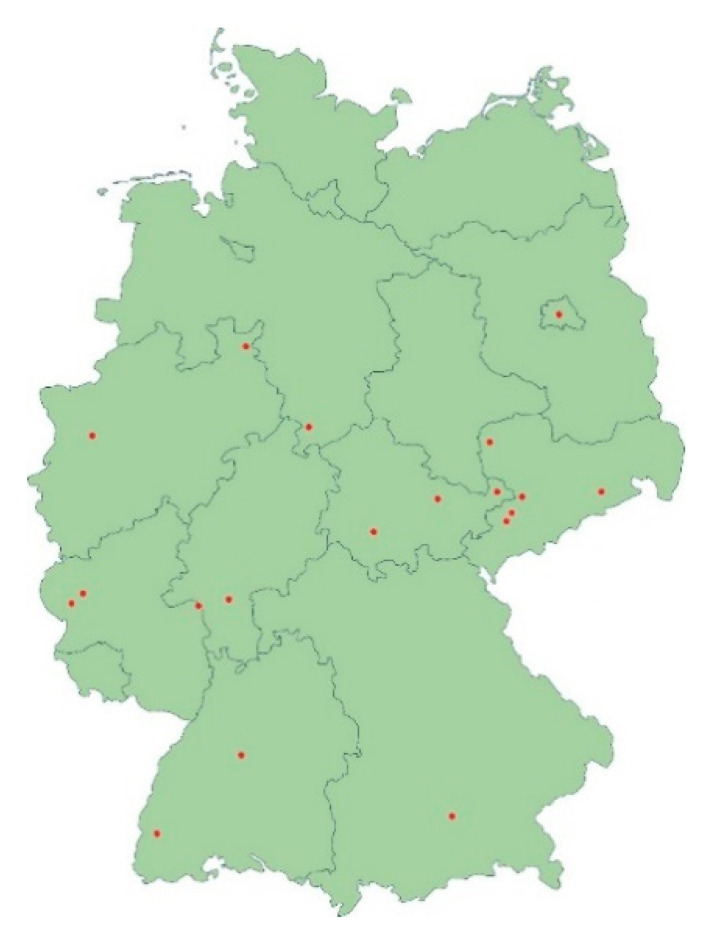
Geographical distribution of patients with dermatophytoses due to the Indian genotype of *T*. *mentagrophytes* identified in this study. Strains of *T*. *mentagrophytes* Type VIII were isolated in nearly all parts of Germany, in large cities like Berlin, Munich and Leipzig as well as in small towns and rural areas.

Numerous reports and findings from our own laboratory suggest that the frequently terbinafine-resistant dermatophyte *T*. *mentagrophytes* Type VIII is becoming increasingly prevalent in countries other than Germany and beyond. *T*. *mentagrophytes* Type VIII has been isolated from skin scrapings of patients in Iraq, Switzerland, Cambodia, Finland, Estonia and Poland [32,33]. We have isolated only individual strains of *T*. *mentagrophytes* Type VIII since 2016, albeit initially under a different name of the species, owing to the classification at that time. There has been a significant increase in the frequency of detecting *T*. *mentagrophytes* Type VIII in the past two years in Germany and we regularly see a strain of *T*. *mentagrophytes* type VIII in routine diagnostics about every two to three weeks, which appears significant. The isolates of *T*. *mentagrophytes* Type VIII originate from all over Germany. Patients with dermatophytoses, due to the Indian genotype of *T*. *mentagrophytes*, have been identified in large cities like Berlin, Munich and Leipzig, as well as small towns and rural areas of Germany (Figure 6).

In retrospect we feel that patients of chronic dermatophytoses caused by *T. mentagrophytes* Type VIII, as seen here, have existed in Germany for years, though in smaller numbers. This genotype seems to be isolated preferentially in migrant patients. Many originally hail from the Indian subcontinent, including India, Bangladesh and Pakistan, but also from Arab countries such as Saudi Arabia, Iraq and Libya. The infection is found to be relatively easily transmitted within the family, especially to the spouse and partner.

### 4.3. Antifungal Resistance In Vitro and Point Mutation Analysis of the Squalene Epoxidase Gene

A significant percentage of the original Indian *T*. *mentagrophytes* strains was resistant to terbinafine both in vitro and due to genetic point mutations in the SQLE gene. Some strains were also found to be partially resistant against itraconazole and voriconazole. Several single point mutations in the fungal SQLE gene, which encodes the target for terbinafine, have also been recorded in *T. rubrum* and *T. mentagrophytes/T. interdigitale*. These mutations have led to substitutions at one of the four amino acid positions Leu^393^, Phe^397^, Phe^415^ and His^440^, and have been associated with terbinafine resistance [12,34]. 71% of isolates of *T. mentagrophytes* Type VIII from India were found to be resistant to terbinafine [15]. The amino acid substitution Phe^397^Leu in the squalene epoxidase of resistant *T*. *mentagrophytes* was found to be highly prevalent (91%) [15]. Two novel substitutions in resistant *Trichophyton* strains isolated in our currently published epidemiological study in India, Ser^395^Pro and Ser^443^Pro, were detected. In contrast, a missense substitution, Ala^448^Thr, was found in terbinafine-sensitive and resistant isolates. Among the 29 strains isolated in Germany, 13 strains (45%) were terbinafine-resistant with breakpoints > 0.2 µg/mL. The other 16 strains showed normal terbinafine susceptibility in vitro against terbinafine with breakpoints < 0.2 µg/mL. It is not clear if all cases of terbinafine resistance occurred after long-term treatment with the drug, or if they had primary resistances. Some patients with resistance were pretreated by oral or topical terbinafine; unfortunately, because the biggest part of our patient´s data on pretreatment were not available. Indeed, however, transmission from affected people to family members who were definitely not pretreated, occurred.

It has been observed that despite in vitro susceptibility, there is often a poor clinical response to terbinafine, as seen in a significant number of patients reported herein. There are some indications of a lack of in vivo correlation of in vitro resistance in dermatophytosis [35]. On the other hand, it was demonstrated that the odds of achieving a cure with terbinafine MIC  <  1 µg/mL strains were 2.5 times the odds of achieving a cure with strains exhibiting MIC ≥ 1 µg/mL, suggesting a good in vitro and in vivo correlation [36]. Further studies are recommended to understand this complex problem of the in vitro/in vivo discordance.

We demonstrated that in vitro resistance to triazoles cannot only be observed in *T*. *mentagrophytes* Type VIII isolated in India, but also in strains from patients residing in Germany. Remarkably, three out of four strains showing resistance to voriconazole were also resistant to itraconazole, strengthening evidence that these strains share a common mechanism of resistance against triazoles [15]. Triazole resistance was recently associated with the substitution Ala^448^Thr in squalene epoxidase [15,30] and such a tendency could be observed in the current collection of strains. However, statistical significance could not be proven due to the relatively small sample size. Further investigations, especially on the role of SQLE double mutants on antifungal susceptibility [30], need to be carried out.

### 4.4. Treatment of Chronic Recalcitrant Dermatophytoses Due to T. mentagrophytes Type VIII

Patients described in this study represented the first reports on an infection due to a terbinafine-resistant *T*. *mentagrophytes* strain of the ITS genotype VIII from Germany and the Indian subcontinent. We aimed to highlight the recalcitrance to even long-term oral and topical treatment with terbinafine observed in our patients of tinea corporis and tinea cruris caused by *T*. *mentagrophytes* Type VIII. Unlike the scenario reported from India, the disease in German patients, seemed to respond to simple topical antifungal therapy other than terbinafine, as exemplified in the case of the baby originating from Bahrain with extensive tinea corporis [17]. It is interesting to note that the child showed significant improvement in the lesions after only one week of local treatment with miconazole and ciclopirox olamine, finally leading to resolution of all lesions with the same topical therapy for a total of four weeks. However, the lesions recurred after reaching Bahrain, where they stopped applying the topical antifungal agents. This case scenario has been experienced by several other patients with chronic, refractory dermatophytoses caused by *T*. *mentagrophytes* Type VIII. Treatment included topical ciclopirox olamine plus miconazole, sertaconazole or luliconazole with patients reporting more benefit with creams containing newer topical azoles. Many such creams are, unfortunately, not approved for use in Germany and other countries in Europe [37]. It seems appropriate to treat such cases with an oral antifungal agent like itraconazole in its adult dose of 100 mg twice daily after a meal for at least four to eight weeks. Some dermatologists recommend higher doses of the drug for a longer duration in widespread disease in patients who have abused topical steroid antifungal combinations for long periods.

## Figures and Tables

**Table 1:** Overview of the 29 patients with dermatomycoses due to *T*. *mentagrophytes* of (ITS) genotype VIII (India) diagnosed all over Germany from 2016–2020. Abbreviations: SQLE, squalene epoxidase; ITS, internal transcribed spacer; TEF1-α, translation elongation factor 1-α; MIC, minimum inhibitory concentration; NCBI, National Center for Biotechnology Information, Bethesda, Maryland; DSM, Deutsche Sammlung von Mikroorganismen und Zellkulturen (German Collection of Microorganisms and Cell Cultures), Braunschweig, Germany; f, female; m, male.

	Strain Number	Collection No. DSMZ	Gender	Age in Years/ Months	Pathology	Additional Information/ Remarks	MIC Terbinafine [µg/mL]	Drug Resistance to Terbinafine	Amino Acid Substitution within the Squalene Epoxidase	Codon Change in SQLE	MIC itraconazole [µg/mL]	Drug Resistance to Itraconazole	MIC Voriconazole [µg/mL]	Drug Resistance to Voriconazole	GenBank Accession Number ITS Gene	GenBank Accession Number TEF1-α Gene (NCBI)	GenBank Accession Number SQLE Gene (NCBI)	Sample Date
1	214677/16	DSM 108903	f	35	Tinea pedis	Indian	0.2	resistant	Phe397Leu	TTC → CTC	0.06	sensitive	0.03	sensitive	MT330252	MT340499	MT700499	2016
2	216377/17	DSM 108902	f	29	Tinea	Foreigners	0.2	resistant	Phe397Leu	TTC → CTC	0.06	sensitive	0.03	sensitive	MT330249	MT340500	MT700500	2017
3	211542/18	DSM 108901	m	19	Tinea corporis	Asylum office Herford	8	resistant	Phe397Leu	TTC → CTC	0.087	sensitive	0.088	sensitive	MT330250	MT340501	MT700501	2018
4	211564/18	DSM 108900	f	30	Tinea unguium et corporis	Indian	<0.2	sensitive	Ala448Thr	GCT → ACT	0.067	sensitive	0.088	sensitive	MT330248	MT340502	MT700502	2018
5	218160/18	DSM 108899	m	35	Tinea corporis et cruris	Indian	<0.2	sensitive	wild type	-	0.125	sensitive	0.03	sensitive	MT330253	MT340503	MT700503	2018
6	218360/18	DSM 108898	f	25	Tinea corporis	married couple on a pilgrimage in Saudi Arabia	<0.2	sensitive	Ala448Thr	GCT → ACT	0.5	resistant	0.5	resistant	MT330255	MT340504	MT700504	2018
7	218691/18	DSM 108897	m	58	Tinea faciei	Visit India	8	resistant	Phe397Leu	TTC → CTC	0.125	sensitive	0.03	sensitive	MT330254	MT340505	MT700505	2018
8	901538/18	DSM 108896	m	4	Tinea corporis	German name	<0.2	sensitive	Ala448Thr	GCT → ACT	0.25	sensitiv	0.25	resistant	MT330251	MT340506	MT700506	2018
9	200618/19	DSM 109024	f	6 months	Tinea corporis	Family came from Bahrain to visit Germany	0.2	resistant	Phe397Leu	TTC → TTA	not applied		not applied		MT330285	MT345568	MT700507	2019
10	202953/19	DSM 109747	m	27	Tinea	Libyan, for three years in Germany, German girlfriend also affected	8	resistant	Phe397Leu	TTC→CTC	0.5	resistant	0.25	resistant	MT330284	MT340507	MT700508	2019
11	203513/19	DSM 109748	f	25	Tinea cruris et inguinalis	Pakistani	<0.2	sensitive	wild type	-	0.0312	sensitive	0.0312	sensitive	MT330280	MT340508	MT700509	2019
12	600174/19	DSM 109749	f	34	Tinea corporis	Indian, was in India when the skin symptoms started	<0.2	sensitive	Ala448Thr	GCT → ACT	0.125	sensitive	0.0312	sensitive	MT330279	MT340517	MT700510	2019
13	205666/19	DSM 109751	m	32	Tinea cruris	Iraqi couple (see no. 14)	<0.2	sensitive	Ala448Thr	GCT → ACT	0.125	sensitive	0.125	sensitive	MT330281	MT340516	MT700511	2019
14	205667/19	DSM 109750	f	31	Tinea corporis	Iraqi couple (see no. 13)	<0.2	sensitive	Ala448Thr	GCT → ACT	0.5	resistant	0.5	resistant	MT330283	MT340515	MT700512	2019
15	208737/19	DSM 110678	f	29	Tinea corporis	Indian name	8	resistant	Phe397Leu	TTC → TTA	not applied	-	not applied	-	MT328783	MT340514	MT700513	2019
16	209934/19	DSM 110677	m	28	Tinea corporis gluteal	Indian name	<0.2	sensitive	Ala448Thr	GCT → ACT	not applied	-	not applied	-	MT330278	MT340513	MT700514	2019
17	600231/19	DSM 110676	m	40	Tinea corporis	Family, large plaques	16	resistant	Leu393Phe	TTA → TTC	not applied	-	not applied	-	MT330282	MT340512	MT700515	2019
18	214174/19	DSM 110675	m	37	Tinea faciei and corporis: cheeks, neck, forehead, and ear helix	German	8	resistant	Phe397Leu Ala448Thr	TTC → CTC GCT → ACT	not applied	-	not applied	-	MT330289	MT340511	MT700516	2019
19	216532/19	DSM 110674	m	27	Tinea corporis	Indian	<0.2	sensitive	Ala448Thr	GCT → ACT	not applied	-	not applied	-	MT330288	MT340510	MT700517	2019
20	217201/19	DSM 110673	m	24	Tinea corporis	Indian	<0.2	sensitive	Val444 Ile Ala448Thr	GTA → ATAGCT → ACT	not applied	-	not applied	-	MT330286	MT340509	MT700518	2019
21	218676/19	-	m	20	Tinea corporis	Foreigner	<0.2	sensitive	wild type	-	not applied	-	not applied	-	MT330256	MT340518	MT700519	2019
22	219238/19	-	f	27	Tinea corporis	Pakistani	<0.2	sensitive	wild type	-	not applied	-	not applied	-	MT330290	MT340519	MT700520	2019
23	600380/19	-	-		Tinea	Indian	<0.2	sensitive	wild type	-	not applied	-	not applied	-	MT330291	MT340520	MT700521	2019
24	220575/19	-	f	24	Tinea corporis gluteal	Student from Bangladesh	<0.2	sensitive	wild type	-	not applied	-	not applied	-	MT330287	MT340521	MT700522	2019
25	600002/20	-	m		Tinea cruris	German patient with stay in Thailand and India	16	resistant	Phe397Leu	TTC → CTC	not applied	-	not applied	-	MT333227	MT340522	MT700523	2020
26	101549/20	-	m	30	Tinea	Foreigner	16	resistant	Phe397Leu Ala448Thr	TTC → CTCGCT → ACT	not applied	-	not applied	-	MT333225	MT340523	MT700524	2020
27	200874/20	-	m	27	Tinea corporis et manum	Foreigner	<0.2	sensitive	wild type		not applied	-	not applied	-	MT333228	MT345569	MT700525	2020
28	900138/20	-	m	51	Tinea corporis	Foreigner	16	resistant	Phe397Leu	TTC → CTC	not applied	-	not applied	-	MT333226	MT340524	MT700526	2020
29	204532/20	-	f	23	Tinea corporis	Originating and migrating for studying from Bangladesh, her father also affected. She was pretreated in her home country by oral voriconazole, without success.	0.2	resistant	Phe397Leu	TTC → CTC	not applied	-	not applied	-	MT333242	MT340525	MT700527	2020

**Table 2:** Reference strains and clinical isolates used to generate the phylogenetic tree based on sequencing of the ITS region of rDNA and the TEF1-α gene. GenBank accession numbers of the nucleotide sequences used in this study are available at the NCBI. Abbreviations: NCBI, National Center for Biotechnology Information, Bethesda, Maryland; ITS, internal transcribed spacer; rDNA, ribosomal DNA; TEF1-α, translation elongation factor 1-α; CBS, Centraal Bureau voor Schimmelcultures (today, Westerdijk Fungal Biodiversity Institute, Utrecht, The Netherlands); DSM, *T. mentagrophytes* Deutsche Sammlung von Mikroorganismen und Zellkulturen (German Collection of Microorganisms and Cell Cultures), Braunschweig, Germany; ATCC, American Type Culture Collection (Manassas, VA, USA). * means a subtype of the genotype II.

Species/Genotype	Strain	Collection	Accession No. NCBI ITS	Accession No. NCBI TEF1-α
*T. interdigitale* ITS genotype I	208223/17	DSM 108620	MK447595	MK460538
*T. interdigitale* ITS genotype I deposited in the NCBI as T. interdigitale	Isolate 2		KC595991	
*T. interdigitale* ITS genotype II	200070/17	DSM 108621	MK447596	MK460539
*T. interdigitale* ITS genotype II deposited in the NCBI as T. interdigitale		RCPF-1301	KP308373	
*T. interdigitale* ITS genotype II deposited in the NCBI as T. interdigitale		CBS 475.93	MF926357	
*T. interdigitale* ITS genotype II deposited in the NCBI as T. interdigitale		CBS 428.63	NR_144900	
*T. interdigitale* ITS genotype II deposited in the NCBI as *T. mentagrophytes*		CBS 647.73	KT155955	
*T. mentagrophytes* deposited in the NCBI as *T. mentagrophytes*		CBS 102.68		KM678062
*T. interdigitale* ITS genotype II *	212063/17	DSM 108905	MK630684	MK751367
*T. interdigitale* ITS genotype II *	250016/18		MN886818	MN886231
*T. mentagrophytes*		CBS 116916		KM678130
*T. interdigitale*		CBS 130940		KM678173
*T. mentagrophytes* ITS genotype III	217704/15	DSM 108630	MK450325	MK460541
*T. mentagrophytes* ITS genotype III	200002/16	DSM 103451	KX866689	MK460540
ITS genotype III deposited in the NCBI as *T. cf. mentagrophytes*		ATCC 60612	KJ606099	
*T. mentagrophytes* ITS genotype III deposited in the NCBI as *T. mentagrophytes*		RCPF-1207	KT253559	
*T. mentagrophytes* ITS genotype III *	217907/15	DSM 108628	MK447605	MK460542
*T. mentagrophytes* ITS genotype III *	218893/16	DSM 108629	MK447604	MK460543
*T. mentagrophytes* ITS genotype III *	900120/17	DSM 108632	MK447606	MK460544
*T. mentagrophytes* ITS genotype III * deposited in the NCBI as *T. interdigitale*		CZE 4473	LN736306	
*T. mentagrophytes* ITS genotype IV	200602/17	DSM 108631	MK447607	MK467447
*T. mentagrophytes* ITS genotype IV	200617/17	DSM 108627	MK447608	MK467446
*T. mentagrophytes* ITS genotype IV	204543/17	DSM 108626	MK447609	MK467445
*T. mentagrophytes* ITS genotype IV deposited in the NCBI as *T. mentagrophytes*		CBS 304.38	MF926360	
*T. mentagrophytes* ITS genotype IV deposited in the NCBI as T. interdigitale		SJEK 4836	FM986773	
*T. mentagrophytes* ITS genotype V deposited in the NCBI as *T. cf. mentagrophytes*		ATCC 46950	KJ606098	
*T. mentagrophytes* ITS genotype V	600014/20		MT374268	MT375511
*T. mentagrophytes* ITS genotype V	600024/20		MT374269	MT375512
*T. mentagrophytes* ITS genotype V	600184/19		MT374259	MT375508
*T. mentagrophytes* ITS genotype V	600197/19		MT374258	MT375509
*T. mentagrophytes* ITS genotype V	600316/19		MT374257	MT375510
*T. mentagrophytes* ITS genotype VI deposited in the NCBI as *T. mentagrophytes*	D15P161/17		MK722518	-
*T. mentagrophytes* ITS genotype VII	210363/16	DSM 108625	MK450323	MK467450
*T. mentagrophytes* ITS genotype VII	218904/16	DSM 108622	MK450322	MK467448
*T. mentagrophytes* ITS genotype VII	200128/17	DSM 108623	MK447611	MK460545
*T. mentagrophytes* ITS genotype VII	215003/16	DSM 108624	MK450324	MK467449
*T. mentagrophytes* ITS genotype VII deposited in the NCBI as *T. mentagrophytes*		NBRC 5809	JN134101	
*T. mentagrophytes* ITS genotype VIII	211509/17	DSM 107597	MH791420	MH802491
T. mentagrophytes ITS genotype VIII	200095/18	DSM 107602	MH791425	MH802496
T. mentagrophytes ITS genotype VIII deposited in the NCBI as *T. interdigitale*	VPCI 390/P/17	-	MH990852	
*T. mentagrophytes* ITS genotype IX	214691/17	DSM 108357	MK447613	MK467444
*T. mentagrophytes* ITS genotype XXV	218292/17	-	MN886815	MN886229
*T. mentagrophytes* ITS genotype XXV	201341/18	-	MN886816	MN886230
*T. quinckeanum*	218251/16			
*T. quinckeanum*	210314/16			
*T. quinckeanum*		ATCC 32457	KJ606088	
*T. quinckeanum*	216686/15		KY680503	KY680502
